# Epidemiological Analysis of Otorhinolaryngologic Pathologies Among Seafarers: A Retrospective Study Based on C.I.R.M. Telemedical Assistance Data, 2023–2025

**DOI:** 10.3390/jpm16090483

**Published:** 2026-09-18

**Authors:** Getu Gamo Sagaro, Carmelo Guarneri, Francesco Amenta

**Affiliations:** 1 Research Department, Centro Internazionale Radio Medico (C.I.R.M.), 00144 Roma, Italy; guarneri@cirm.it (C.G.); presidenza@cirm.it (F.A.); 2 School of Public Health, College of Health Sciences and Medicine, Wolaita Sodo University, Wolaita Sodo 138, Ethiopia

**Keywords:** seafarers, otorhinolaryngology, ear, nose and throat (ENT) pathologies, occupational health, epidemiology

## Abstract

**Background**: Seafarers are exposed to a variety of occupational and environmental factors that may influence their individual risk of developing otorhinolaryngologic (Ear, Nose and Throat, ENT) conditions while at sea. Understanding the distribution of ENT-related health problems in this population may inform personalized approaches to prevention, early detection, and remote healthcare management. However, epidemiological information on ENT-related medical consultations among seafarers remains limited. This study aimed to describe the distribution of ENT conditions among seafarers receiving telemedicine assistance from Centro Internazionale Radio Medico (C.I.R.M.), providing insights for individualized healthcare strategies in maritime populations. We also compared demographic and occupational characteristics of ENT and non-ENT medical assistance cases. **Methods**: A retrospective descriptive study was conducted using C.I.R.M. telemedical assistance records for seafarers who sought medical assistance between 1 January 2023 and 31 December 2025. Cases diagnosed with ENT conditions were identified and analyzed according to age, gender, nationality, occupational rank, worksite, vessel type, and diagnostic category. The frequency and distribution of ENT consultations were described using descriptive statistics. Comparisons between ENT and non-ENT cases were performed using Pearson’s chi-square test, with statistical significance set at *p* < 0.05. **Results**: A total of 17,897 telemedical-assisted cases were recorded during the study period, of which 751 (4.2%) involved ENT conditions. The most common ENT consultations were ear symptoms and inflammation (No. = 286, 38.0%) and tonsillopharyngitis (No. = 180, 24.0%). Among ENT cases, the largest proportion occurred among seafarers aged 30–39 years (39.3%), deck crew members (30.4%), personnel working in the deck department (48.7%), and non-European seafarers (50%). Significant differences were observed between ENT and non-ENT cases according to age group (χ^2^ = 16.58, *p* = 0.001), occupational rank (χ^2^ = 11.14, *p* = 0.025), and nationality (χ^2^ = 82.76, *p* < 0.001). **Conclusions**: ENT conditions accounted for a small but noteworthy proportion of telemedical assistance cases among seafarers, with significant variations according to age, rank, and nationality. These findings highlight population subgroups with different healthcare needs and may contribute to more personalized preventive and telemedical strategies for seafarers.

## 1. Introduction

Seafarers are a unique occupational group, exposed to a wide range of environmental, occupational, and health risks while working at sea [1,2]. Prolonged periods onboard vessels, limited access to healthcare facilities, demanding work schedules, noise, vibration, changing climates, and occupational hazards can contribute to the development of various medical conditions that require remote medical assistance [1,3,4]. Medical problems must be recognized and managed promptly in the isolated nature of maritime operations to maintain crew health and safety and ensure the vessel’s uninterrupted operation.

Telemedical assistance services play a relevant role in supporting seafarers who develop acute illnesses or injuries during voyages. Several studies have shown that cardiovascular diseases, gastrointestinal disorders, injuries, musculoskeletal conditions, and respiratory illnesses are among the most common reasons for seeking medical attention at sea. Otorhinolaryngologic (Ear, Nose and Throat, ENT) conditions represent another category of health problems that may affect a seafarer’s fitness for duty, performance at work, communication capabilities, and general health. Otorhinolaryngologic pathologies encompass a variety of disorders involving the ear, nose, and throat, including acute infections, trauma, epistaxis, vestibular disorders, and other conditions that may require urgent medical evaluation and treatment. Maritime occupational exposures such as continuous noise, changes in atmospheric pressure, humidity, airborne irritants, and workplace accidents may increase the risk of certain ENT-related conditions among seafarers. On the other hand, the limited availability of medical facilities on board vessels may complicate the management of these conditions that require telemedical consultations or medical referrals [5,6,7].

The epidemiological characteristics of ENT pathologies among seafarers remain understudied despite the importance of occupational health in the maritime sector. Most published studies have focused on broader categories of diseases and injuries, with limited attention given specifically to otorhinolaryngologic conditions [3,7]. Accordingly, there is a lack of information on the distribution of ENT issues by demographic and occupational characteristics, such as age, gender, rank, and worksite on board vessels. In the maritime setting, where access to specialist care is often limited and telemedicine plays a central role in clinical decision-making, understanding how ENT conditions vary across demographic and occupational groups may support more individualized risk assessment, preventive strategies, and remote clinical management. Also, identifying occupational groups that are more often affected may assist institutional actors, shipping companies, maritime healthcare providers, and telemedical assistance services in developing targeted interventions and educational programs aimed at reducing the burden of these conditions.

The purpose of this study was to analyze the epidemiological characteristics of otorhinolaryngologic pathologies among seafarers who received telemedical assistance from Centro Internazionale Radio Medico (C.I.R.M.) between 2023 and 2025. C.I.R.M. is the Italian Telemedical Maritime Assistance Service (TMAS). This work was specifically intended to determine the proportion of otorhinolaryngologic pathologies among all telemedical assistance cases and to evaluate their distribution according to age, sex, rank, nationality, vessel type, and onboard worksite category to inform general preventive strategies and more personalized approaches to maritime healthcare.

## 2. Materials and Methods

This retrospective descriptive epidemiological study was conducted using the telemedical assistance records of the C.I.R.M. database. For over 91 years, C.I.R.M. has provided telemedical assistance to seafarers worldwide regardless of vessel location, flag state, or crew nationality [7]. C.I.R.M. provides remote medical consultations in support of maritime medical management. It maintains a comprehensive database of medical assistance requests. This study has analyzed medical assistance cases recorded between 1 January 2023 and 31 December 2025. Anonymized data relevant to the study objectives were extracted from the C.I.R.M. database following authorization to use the database. The extracted dataset included demographic, occupational, and clinical information about seafarers who received telemedical assistance during the study period.

### 2.1. Study Population and Data Collection

The study population consisted of individual seafarers who received telemedical consultations during the study period. Each seafarer was included only once in the analysis, regardless of how many telemedical consultations they received. Repeated consultations by the same seafarer were not counted as separate cases, ensuring that the analysis was based on unique individuals. According to the medical diagnoses recorded in the database, otorhinolaryngologic pathologies were identified. C.I.R.M. physicians diagnosed ENT conditions using various telemedicine modalities, and cases were classified by the main presenting symptom or clinical sign that prompted the patient to seek medical care. The identified cases were recorded in a database using the World Health Organization’s International Statistical Classification of Diseases and Related Health Problems, 10th Revision (WHO ICD-10). Diagnoses were classified using the relevant ICD-10 codes: ear and mastoid process (H60–H95), upper respiratory tract and related otorhinolaryngologic conditions (J00–J05 and J30–J39), and selected injuries and foreign bodies involving ENT structures (S00–S19 and T16–T19). Cases involving otorhinolaryngologic conditions of the ear, nose, throat, or related structures were eligible for consideration. A standardized data collection form was used to extract the data. Demographic variables included age and gender. The age categories were <30 years, 30–39 years, 40–49 years, and ≥50 years. Occupational characteristics included rank and onboard worksite. Rank was classified into officers (deck officers and engine officers) and non-officers (deck crew, engine crew, and catering staff). The workplace was categorized by the ship’s area where the seafarer primarily worked, namely the deck, engine, and catering departments. Nationality was recorded in the C.I.R.M. database based on the seafarer’s reported country of nationality. For analysis, countries were categorized as European or non-European according to their geographic location. This European/non-European classification was used as a demographic variable in the comparative analyses. Clinical information on the diagnosis of the ENT conditions was then collected and classified into different groups.

### 2.2. Statistical Analysis

Data were checked, recoded, entered, and analysed using the R programming language (Version 4.4.1) [8]. Descriptive statistics were used to summarize the study findings. The frequencies and percentages of categorical variables were presented, whereas the means and standard deviations were used to summarize continuous variables. The proportion of ENT issues among all medical assistance cases was calculated. The distribution of ENT pathologies by demographic and occupational characteristics was presented in contingency tables. Chi-square or Fisher’s exact tests, as appropriate, were used to assess differences in the distribution of categorical variables between ENT-related and non-ENT medical assistance cases. Cases with unknown or missing values were excluded from the analysis of the corresponding variable. The number of observations included in each comparison varied according to data availability. We considered a two-sided *p*-value of less than 0.05 statistically significant.

## 3. Results

During the study period, a total of 17,897 seafarers received medical assistance from C.I.R.M. The annual number of consultations increased gradually, with 5570 cases recorded in 2023, 5879 in 2024, and 6448 in 2025. Among all medical assistance requests, 751 cases (4.2%) were related to ENT conditions. The average age of seafarers with otorhinolaryngologic symptoms was 38.37 (SD = 10.56). The number of ENT consultations increased slightly during the study period, accounting for 235 cases in 2023, 238 cases in 2024, and 278 cases in 2025. These findings indicate a relatively stable burden of ENT-related conditions among seafarers requiring telemedical assistance.

### 3.1. Distribution of ENT Diagnoses

The distribution of ENT diagnoses is presented in Table 1. Ear-related conditions accounted for most cases. The most common diagnostic category was ear symptoms and inflammation, comprising 286 cases (38.0%), including otalgia (200 cases, 69.9%), ear fullness (50 cases, 17.5%), and nonspecific inflammatory ear conditions (36 cases, 12.6%). Tonsillopharyngitis was the second most frequent diagnosis, comprising 180 cases (24.0%), followed by acute/middle ear infections with 101 cases (13.5%). Other upper respiratory tract infections (URTIs), including pharyngitis, rhinitis, sinusitis, and laryngitis, accounted for 81 cases (10.8%). Less frequent diagnoses included hearing and vestibular disorders (32 cases, 4.3%), other ENT conditions (28 cases, 3.7%), acute otitis externa (25 cases, 3.3%), and ENT trauma (18 cases, 2.4%). Overall, ear-related disorders, including infections, inflammatory conditions, hearing disturbances, vestibular disorders, and trauma, were the predominant reason for ENT consultations, whereas throat-related conditions, particularly tonsillopharyngitis, constituted the second largest diagnostic group (Table 1).

### 3.2. Demographic and Occupational Characteristics of Seafarers Suffering from ENT Pathologies

Most participants were aged 30–39 years (No. = 295, 39.3%), followed by those aged 40–49 years (No. = 162, 21.6%). The study population was predominantly male, comprising 737 seafarers (98.0%), while females accounted for 14 seafarers (2.0%). In terms of occupational rank, deck crew represented the largest group (228, 30.4%), followed by engine crew (145, 19.3%), deck officers (138, 18.4%), engine officers (127, 17.0%), and galley personnel (63, 8.4%). Regarding nationality, 375 participants (50.0%) were non-European, and 229 (30.5%) were European. Nationality data were unavailable for 147 participants (19.5%). In terms of worksite, most consultations originated from the deck department (366, 48.7%), followed by the engine department (272, 36.2%). Regarding vessel type, most seafarers who sought medical advice for ENT conditions were serving on cargo ships (317, 42.2%), followed by tankers (256, 34.0%) and bulk carriers (113, 15.0%) (Table 2).

### 3.3. Comparison of ENT and Non-ENT Medical Assistance Cases

Cases with unknown demographic or occupational information were excluded from the chi-square analyses to ensure valid comparisons between ENT and non-ENT medical assistance cases. Percentages were calculated using cases with available data for each variable. Accordingly, a total of 742 ENT cases and 17,022 non-ENT cases with available age information were included in the analysis. Age distribution differed significantly between ENT and non-ENT medical assistance cases (χ^2^ = 16.58, *p* = 0.001). Among ENT cases, the largest proportion occurred in seafarers aged 30–39 years (39.8%), followed by those aged 40–49 years (21.8%). Compared with non-ENT cases, ENT conditions were proportionally more common among younger seafarers, whereas non-ENT conditions were relatively more frequent among older age groups. In terms of rank group, 701 ENT cases and 15,817 non-ENT cases with available rank information were included in the analysis. Rank distribution differed significantly between ENT and non-ENT medical assistance cases (χ^2^ = 11.14, *p* = 0.025). Deck crew constituted the largest proportion of both ENT (32.5%) and non-ENT cases (36.3%). Compared with non-ENT cases, ENT cases were proportionally more common among officers [deck officers (19.7% vs. 15.4%) and engine officers (18.1% vs. 17.4%)].

No significant difference was observed in worksite distribution between ENT and non-ENT medical assistance cases (χ^2^ = 0.08, *p* = 0.962). In both groups, the majority of cases occurred among personnel working in the deck department (52.2% vs. 51.7%), followed by the engine department (38.8% vs. 39.0%). Similarly, the distribution of gender and vessel type did not differ significantly between ENT and non-ENT cases, as indicated by the chi-square analyses (gender: χ^2^ = 2.19, *p* = 0.139; vessel type: χ^2^ = 4.14, *p* = 0.247). Nationality distribution differed significantly between ENT and non-ENT cases (χ^2^ = 82.76, *p* < 0.001). European seafarers accounted for a higher proportion of ENT cases compared to non-ENT cases (38.0% vs. 22.0%) (Table 3).

## 4. Discussion

The present study provides an epidemiological assessment of otorhinolaryngologic (ENT) conditions among seafarers requiring C.I.R.M. telemedical assistance from 2023 to 2025. The study evaluated the distribution of ENT consultations among seafarers and compared their demographic and occupational characteristics with those of non-ENT medical assistance cases. We found that ENT conditions accounted for approximately 4.2% of all medical assistance consultations during the study period. Although ENT pathologies represented a relatively small share of the overall caseload, they remain clinically important because they can affect hearing, communication, balance, and operational performance, which could be detrimental to individual health and maritime safety.

As reported in previous studies on seafarer morbidity patterns, gastrointestinal disorders, injuries, dermatological conditions, cardiovascular diseases, musculoskeletal disorders, and respiratory illnesses are the most common reasons for seafarers to seek medical attention [5,6,7,9,10]. TMAS databases have generally been used for studies focusing on these broader disease categories, while ENT conditions have received comparatively little attention. The present study has therefore addressed a gap in the maritime health literature by providing novel information regarding the epidemiological characteristics of ENT-related consultations among seafarers.

In the present study, ear symptoms and inflammation, which comprised 286 cases (38.0%), were the most frequently reported ENT conditions, followed by tonsillopharyngitis, which accounted for 180 cases (24.0%). Biologically, these findings make sense in light of the occupational and environmental conditions encountered at sea. Many factors can contribute to ENT-related symptoms, including humidity, temperature fluctuations, airborne irritants, confined living environments, and occupational hazards. Previous occupational health studies have identified prolonged noise exposure, adverse climatic conditions, and respiratory infections as significant contributors to ENT morbidity. Furthermore, maritime environments can increase the risk of acute ear infections, upper respiratory tract infections, vestibular disorders, and trauma-related ENT conditions.

We found a significant association between age and the occurrence of ENT consultations. Compared with non-ENT cases, ENT consultations were proportionally more common among younger seafarers, particularly those under 30 years of age. Conversely, non-ENT conditions were relatively more prevalent in older age groups. These findings are consistent with previous studies indicating that younger seafarers tend to experience a higher frequency of acute medical conditions and injuries, while older seafarers tend to suffer from chronic diseases such as cardiovascular disorders, hypertension, diabetes, and musculoskeletal conditions [5,11,12,13,14]. Moreover, studies among maritime workers have shown that the burden of chronic diseases, including cardiovascular and lifestyle-related conditions, increases with age, reflecting the cumulative effects of occupational exposures and health-related risk factors over the course of a seafaring career [15,16]. On the other hand, many of the ENT conditions identified in this study, such as acute infections and inflammatory disorders, are acute conditions that may affect younger individuals more frequently. Additionally, the higher proportion of ENT consultations among younger seafarers may be attributable to their greater exposure to operational activities and the environmental conditions associated with their work on board.

There was also a significant association between occupational rank and ENT consultations. Even though deck crew represented the largest proportion of ENT cases, deck officers accounted for a proportionally greater share of ENT consultations than non-ENT cases. It has been demonstrated in previous maritime studies that occupational rank influences both disease patterns and healthcare-seeking behaviour among seafarers. In terms of job responsibilities, work schedules, educational background, and health awareness, officers and crew members often differ. Officers are responsible for navigation, communication, and safety-critical operations, so symptoms such as hearing loss, balance problems, or difficulty communicating verbally may negatively affect their performance. As a result, officers may be more likely to seek medical advice through telemedicine when experiencing ENT-related symptoms. However, the observed differences may also reflect variations in workforce composition and should be interpreted with caution. Seafarers’ nationality was found to be one of the strongest factors associated with ENT consultations. ENT consultations were significantly more prevalent among European seafarers than non-European seafarers, while non-European seafarers accounted for a smaller proportion of ENT consultations. Previous maritime health studies investigating injury patterns, healthcare utilization, and medical consultations among multinational crews have reported similar variations based on nationality. However, the underlying reasons for these differences remain uncertain. There may be differences in healthcare-seeking behaviour, communication with TMAS physicians, cultural perceptions of illness, occupational distribution, and access to onboard medical resources as possible explanations. Nevertheless, the higher proportion of European seafarers among ENT consultations should be interpreted cautiously. This study included only seafarers who sought telemedical assistance, not the total seafarer population. Therefore, findings related to nationality reflect the distribution of ENT consultations within the telemedical service population and cannot be interpreted as evidence of a higher prevalence or risk of ENT conditions among European seafarers. Differences in the demographic composition of the TMAS population, as well as in healthcare-seeking and consultation patterns, may also have contributed to the observed distribution. Rather, they indicate that there are differences in consultation procedures meriting further investigation [5].

Regarding the implications of these findings for personalized medicine, the observed differences in ENT-related telemedical consultations according to demographic and occupational characteristics suggest that healthcare needs may vary among seafarers. Although ENT conditions accounted for a relatively small proportion of all telemedical assistance cases, the identified patterns provide valuable information for telemedical service planning and targeted healthcare strategies. Incorporating individual characteristics, particularly age, occupational rank, and nationality, into clinical assessment may facilitate more tailored preventive measures and telemedical management approaches. Even though the observed patterns of ENT-related telemedical consultations may provide useful insights for preventive planning and the allocation of telemedical resources onboard, they should not be interpreted as evidence of individual-level risk profiles or predictors of ENT disease. Further analytical studies incorporating individual, occupational, and environmental characteristics are needed to identify independent predictors of ENT conditions.

Limitations of the study: Interpreting the findings of this study requires consideration of many limitations. First, the study relied on routinely collected TMAS data, and diagnoses were based on remote medical consultations rather than direct clinical examinations. Second, no information was available regarding occupational exposures, smoking habits, medical history, noise exposure, or the duration of sea service. Third, the study was limited to seafarers who requested medical assistance and, therefore, may not be representative of the prevalence of ENT conditions among all seafarers. Fourth, an incomplete recording of some demographic information in the telemedicine database, particularly nationality, which was unavailable for 19.5% of ENT cases. This may limit the completeness and representativeness of the nationality-specific findings, which should therefore be interpreted with caution. Finally, due to the descriptive and cross-sectional nature of the study, causal relationships between demographic factors or occupational factors and ENT consultations cannot be established. Overall, the findings of the present study reflect telemedical consultation patterns rather than population-level ENT epidemiology among seafarers. Despite the limitations of the present study, it provides important epidemiological evidence regarding ENT-related consultations among seafarers. It was found that ENT conditions constitute a distinct part of maritime healthcare demand and that their distribution differs by age, occupation, and nationality.

## 5. Conclusions

In summary, C.I.R.M. managed a significant proportion of cases involving ENT conditions via telemedical assistance during the study period. The most frequently reported ENT consultations involved ear disorders, especially ear symptoms and inflammation, followed by tonsillopharyngitis, which underscores the importance of treating these conditions in maritime healthcare. The present study found significant differences in the distribution of ENT consultations according to seafarers’ age, occupational rank, and nationality. Compared with non-ENT cases, ENT conditions were proportionally more common among younger seafarers, deck officers, and European seafarers, whereas no significant differences were observed according to onboard worksite, suggesting that ENT conditions may affect seafarers across different shipboard departments. Given the impact of otorhinolaryngologic disorders on hearing, communication, balance, and fitness for duty, early recognition and appropriate management remain essential in maritime populations. By identifying demographic and occupational characteristics associated with different patterns of ENT-related medical assistance, this study provides an epidemiological basis for preventive strategies and more individualized approaches to prevention and telemedical management. These findings contribute to the limited literature on ENT conditions among seafarers and may support maritime health professionals, telemedical assistance services, and shipping companies in developing targeted healthcare strategies. Future studies incorporating detailed clinical diagnoses, occupational exposure assessments, and longitudinal follow-up are needed to further define individual risk factors and improve personalized approaches to ENT care in maritime settings.

## Figures and Tables

**Table 1:** Distribution of ENT Pathologies by Diagnosis among Seafarers, 2023–2025.

ENT Diagnosis	Number of Cases (No. = 751)	Percentage (%)
Acute otitis externa	25	3.3
Acute/middle ear infection	101	13.5
Ear symptoms/inflammation	286	38.0
Hearing/vestibular disorders	32	4.3
Tonsillopharyngitis	180	24.0
Other URTI	81	10.8
ENT trauma	18	2.4
Other ENT conditions	28	3.7

**Table 2:** Characteristics of seafarers with otorhinolaryngologic pathologies, 2023–2025.

Variable	Number of Cases (No. = 751)	Percentage (%)
Age group		
<30	158	21.0
30–39	295	39.3
40–49	162	21.6
≥50	127	17.0
Unknown	9	1.2
Rank		
Deck officer	138	18.4
Engine officer	127	17.0
Deck crew	228	30.4
Engine crew	145	19.3
Galley	63	8.4
Unknown	50	6.7
Gender		
Male	737	98.0
Female	14	2.0
Nationality		
European	229	30.5
Non-European	375	50.0
Unknown	147	19.5
Worksite		
Deck	366	48.7
Engine	272	36.2
Catering	63	8.4
Unknown	50	6.7
Vessel Type		
Cargo ships	317	42.2
Tankers *	256	34.0
Bulk carriers	113	15.0
Other ships	18	2.4
Unknown	47	6.3

*: conventional tankers, LPG tankers, and oil/chemical tankers.

**Table 3:** Comparison of ENT and Non-ENT medical assistance cases among Seafarers, 2023–2025.

Variable	ENT Cases No. (%)	Non-ENT Cases No. (%)	χ^2^	*p*-Value
Age group (years)				
<30	158 (21.3)	2861 (16.8)		
30–39	295 (39.8)	6470 (38.0)		
40–49	162 (21.8)	4016 (23.6)		
≥50	127 (17.1)	3675 (21.6)		
Total	742 (100)	17,022 (100)	16.58	**0.001**
Rank				
Deck officer	138 (19.7)	2435 (15.4)		
Engine officer	127 (18.1)	2756 (17.4)		
Deck crew	228 (32.5)	5748 (36.3)		
Engine crew	145 (20.7)	3421 (21.6)		
Galley	63 (9.0)	1457 (9.2)		
Total	701 (100)	15,817 (100)	11.14	**0.025**
Gender				
Male	737 (98.0)	16,942 (98.8)		
Female	14 (2.0)	204 (1.2)		
Total	751 (100)	17,146 (100)	2.19	0.139
Nationality				
European	229 (38.0)	2826 (22.0)		
Non-European	375 (62.0)	10,039 (78.0)		
Total	604 (100)	12,865 (100)	82.76	**<0.001**
Worksite				
Deck	366 (52.2)	8183 (51.7)		
Engine	272 (38.8)	6177 (39.0)		
Catering	63 (9.0)	1457 (9.2)		
Total	701 (100)	15,817 (100)	0.08	0.962
Vessel Type				
Cargo ships	317 (45.0)	7212 (45.0)		
Tankers *	256 (36.4)	6016 (37.5)		
Bulk carriers	113 (16.0)	2247 (14.0)		
Other ships	18 (2.6)	570 (3.6)		
Total	704 (100)	16,045 (100)	4.14	0.247

*: conventional tankers, LPG tankers, and oil/chemical tankers.

## Data Availability

The datasets used and/or analysed during this study are available upon reasonable request from the corresponding author.
